# Eight Days of Earth Reambulation Worsen Bone Loss Induced by 1-Month Spaceflight in the Major Weight-Bearing Ankle Bones of Mature Mice

**DOI:** 10.3389/fphys.2018.00746

**Published:** 2018-06-25

**Authors:** Maude Gerbaix, Heather White, Guillaume Courbon, Boris Shenkman, Guillemette Gauquelin-Koch, Laurence Vico

**Affiliations:** ^1^French National Center for Space Studies, Paris, France; ^2^INSERM, UMR 1059, University of Lyon, Jean Monnet University, Saint-Étienne, France; ^3^Institute for Biomedical Problems, Russian Academy of Sciences, Moscow, Russia

**Keywords:** microgravity, recovery, ankle bones, Bion-M1, mice, microarchitecture

## Abstract

Spaceflight induces bone alterations with site-specific rates of bone loss according to the weight-bearing function of the bone. For the first time, this study aimed to characterize bone microarchitecture and density alterations of three ankle bones (calcaneus, navicular, and talus) of mice after spaceflight and to evaluate the impact of 8 days of Earth reambulation. Ten C57BL/6N male 4-month-old mice flew on the Bion-M1 biosatellite for 1 month; half were euthanized within 24-h of return and half after 8-days recovery on Earth. Bone microarchitecture and quality was assessed by microtomography (μCT). Whole calcaneus bone volume fraction decreased in Flight group (−6.4%, *p* < 0.05), and worsened in the Recovery group (−11.08%, *p* < 0.01), when compared to Control group. Navicular and talus trabecular bone volume fraction showed trends toward decrease in Flight and differences reached statistical significance in Recovery group (−8.16%; −8.87%, respectively; *p* < 0.05) when compared to Control group. At calcaneus, cortical thickness decreased in Recovery vs. Control groups (−11.69%; *p* < 0.01). Bone surface area, reflecting periosteal bone erosion, significantly increased in all bone sites analyzed. Qualitative analyses of 3-D bone reconstruction revealed local sites of cortical thinning and bone erosion, predominantly at articulations, muscle insertions, and ground contact bone sites. Overall, spaceflight-induced bone loss in ankle bones was site and compartment specific whilst the tissue mineral density of the remaining bone was preserved. Eight days after landing, bone status worsened as compared to immediate return.

## Introduction

Across the body of research for human and rodents, it is well documented that weight-bearing bones are subjected to most adverse effects of spaceflights for both mature skeletons (Vico et al., 2000; Gerbaix et al., 2017) and growing skeletons causing retardation of matrix maturation (Morey and Baylink, 1978; Zernicke et al., 1990; Doty et al., 1992; Cavolina et al., 1997; Evans et al., 1998). However, many non-weight bearing sites remain affected (Harrison et al., 2003; Kohler et al., 2005) although to a lesser extent. Indeed, LeBlanc et al. (2000) found that a monthly BMD change of −0.04% in the arm was much lower than −1.35% observed at the pelvis of humans following 4–14.4 months onboard the Mir space station. Therefore, weight-bearing bones are often of greater interest for studying unloading implications on the structural integrity and thus the biomechanical strength of the bone.

Weight-bearing sites that are most commonly studied, following microgravity exposure, include the tibia (Jee et al., 1983; Collet et al., 1997; Wronski et al., 1998; Vico et al., 2000), femur (Harrison et al., 2003; Keune et al., 2015; Gerbaix et al., 2017), and the femoral neck and greater trochanter region forming the hip (Lang et al., 2004; Blaber et al., 2013). The calcaneus as a weight-bearing bone of both the human and mouse is largely understudied following microgravity exposure. Previously, research of microgravity impact on the ankle focused only on the calcaneus, and assessed only bone mineral density by 2D X-ray imaging (DXA) (Rambaut and Johnston, 1979; Lang et al., 2004) or roughly approached bone integrity with ultrasound attenuation measurements (Collet et al., 1997). The increasing presence of 3D μCT in research laboratories over the last two decades, allows for more readily available sophisticated analysis; which, until now has not been used to assess the microarchitecture, density, and structural integrity of the calcaneus following microgravity exposure. Despite limited research, the calcaneus is still the most studied bone of the foot and ankle following microgravity exposure. Wang et al. (2015) demonstrated that in humans the navicular, in relation to its size, experiences the largest transfer of force in the foot (0.33 × body weight). From this and admitting that human and mouse ankles are comparable (Chang et al., 2016), it could be hypothesized that when unloaded the navicular may endure greatest deterioration. Despite the fact that mice and human ankles might experience different loading patterns, Wang et al. (2015) study highlight the need to consider multiple bones within the ankle region due to differences that exist in loading across the foot. Furthermore, bone recovery in mature mice following microgravity exposure has poorly been reported (Harrison et al., 2003; Gerbaix et al., 2017). For astronauts, bone recovery was predicted to last at least as long as the flight duration in weight-bearing bone sites (Vico et al., 2000; Sibonga et al., 2007). Until recently data had shown that spaceflight-induced bone loss was not rescued 1 year after a 6 month spaceflight (Vico et al., 2017). The observed slow bone recovery may be modulated in the ankle region, due to large involvement in the gait cycle of both mice (Mendes et al., 2015) and humans (Loudon et al., 2008).

For the first time, our study aims to investigate the structural and density changes of the total and cortical bone, in the ankle bones of mature mice by analyzing the calcaneus, navicular, and talus after 1-month spaceflight and an 8-day recovery period post-flight. We also examined the nature of bone loss within each bone by assessing qualitative analysis of 3D reconstructions.

## Materials and Methods

The study protocol was approved by the Institutional Animal Care and Use Committee (IACUC) at the Institute of Mitoengineering, Moscow State University (Protocol Number: 35, 1 November 2012) and the Biomedical Ethics Committee of the Institute of Biomedical Problems (IBMP) (Protocol Number: 319, 4 April 2013). Experimental procedures were performed in compliance with the European Convention for the Protection of Vertebrate Animals used for Experimental and Other Scientific Purposes (Andreev-Andrievskiy et al., 2014).

### Animals

Twenty-eight C57BL/6N male mice (aged 19–20 weeks) were obtained from the Animal Breeding Facility (Branch of Shemyakin & Ovchinnikov Institute of Bioorganic Chemistry, Moscow, Russia). The flight group (Flight, *n* = 5) flew on the 30-day orbital unmanned Bion-M1 biosatellite and was euthanized within 24-h after landing. The recovery group (Recovery, *n* = 5) were exposed to the same 30-day flight on the Bion-M1 biosatellite as the flight group, but recovered for a period of 8-days prior to euthanasia. Two different control groups were used during this study. The habitat control conducted within a climatic chamber (Habitat Control, *n* = 6) that replicated the habitat settings of the flight, including housing, food, temperature, humidity, and gas composition. A standard control group was placed in standard housing conditions (Control, *n* = 12). Control mice were housed, in cohorts of three mice, in standard GM500 individually ventilated cages (*Techniplast, Italy*, floor area 501 cm^2^). The two groups on board the 30-day flight (Flight and Recovery) and Habitat Control group were housed in cylindrical habitats (*Biofizpribor, Saint Petersburg, Russia*). Each unit consisted of five individual habitats, housing three mice, with a cylindrical diameter of 98 and 200 mm length (volume: 153 cm^3^). Selection of stable groups prior to the study and a co-adaptation phase assisted with group organization and allowed necessary rearrangement of 14% of the social groups, for conflict avoidance during each 30-day experiment (Andreev-Andrievskiy et al., 2014). Flight, Recovery, and Habitat Control animals consumed a paste-like diet containing 74.6% of H_2_O and casein gelling agent whereas control mice consumed a standard pellet diet.

### Sample Preparation

Following euthanasia by cervical dislocation 13–24 h after landing for Flight, and 8 days after landing for Recovery, the hind foot and ankle region was dissected from the limb, soft tissue remained *in situ* and stored in a 70% ethanol solution. Right hind feet were positioned uniformly in an airtight cylindrical sample holder, diameter 20 mm, containing 70% ethanol solution. A circular foam pad ensured uniform positioning of the foot with the inferior aspect orientated to the base of the sample holder. Dissection of mice ankles resulted in talus damages to several mice feet. Consequently, for the talus analysis, the Habitat Control group was not available, the Control and Recovery groups were diminished in size (*n* = 6; *n* = 4, respectively), whilst the Flight group remained constant (*n* = 5).

### Microtomography

**FIGURE 1:**
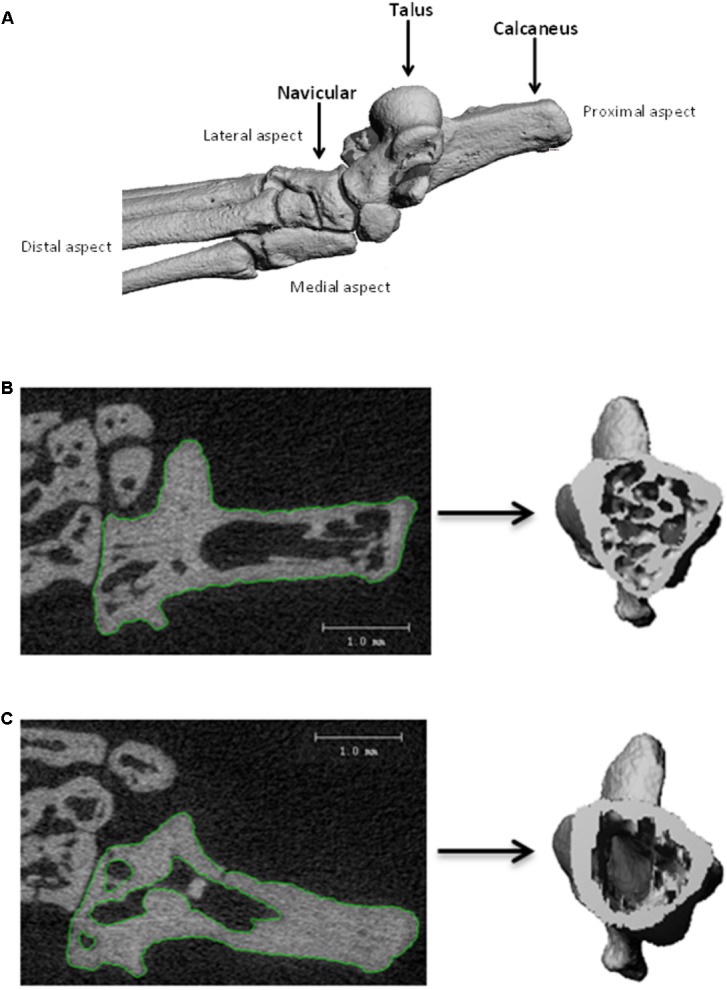
Three-dimensional reconstructions of the ankle bones **(A)**. Contouring of 2D projection images to produce 3D reconstruction, for: **(B)** single contouring for total bone analysis; **(C)** double contouring for cortical bone analysis.

Hind feet were scanned by employing μCT using the VivaCT 40 (Scanco Medical AG, Bassersdorf, Switzerland) for analysis of bone microarchitecture parameters and volumetric density. Experimental parameters used for imaging were as follows: 55 kV peak voltage, 145 μA current, 10.5 μm voxel size, collecting 2000 projections each rotation at 250 ms integration time. A gantry used by the VivaCT 40 allowed the sample to remain stationary whilst the microfocus x-ray tube, scintillator, and photodetector rotated around the sample holder; for the acquisition of 266–454 microtomographic slices at increments of 10.5 μm. Three dimensional reconstruction of the ankle is given on **Figure 1A**. Calcaneus, navicular, and talus bones were analyzed for both the total and cortical bone using the μCT Evaluation Program V6.5-3 (Scanco Medical AG, Bassersdorf, Switzerland). The approach taken for total bone analysis was single contouring of the region of interest captured within the high resolution 2D projections (**Figure 1B**). Cortical bone analysis utilized double contouring (**Figure 1C**). The following segmentation parameters (gauss sigma/gauss support/threshold) were utilized for 3D reconstructions: total bone analysis 0.8/1.0/335; cortical bone analysis 0.8/1.0/260. Threshold determination for total bone analysis was established using the integer that best differentiated between bone (white) and non-bone (black) areas. Integer determination was completed for one sample from control, habitat, flight, and recovery groups, thus the average threshold for total bone analysis was applied. Cortical bone analysis used appropriate predetermined segmentation parameters. Three-dimensional reconstructions provided the operator with an image for qualitative and quantitative analysis parameters. The parameters that were analyzed are outlined in **Table 1** in relation to use for total and cortical analysis and in accordance to the American Society for Bone and Mineral Research recommendations (Bouxsein et al., 2010). Total analysis denotes the two types of bone: cortical and trabecular. Contouring performance was validated against a second operator prior to completion. Inter-individual coefficient of variation (CV = SD/mean) is reported in the results. Completion of contouring by one assessor ensured congruency of measurements and consistency during the analysis procedure. In order to ensure reproducibility of results, i.e., test–retest reliability, the one assessor completed four measurements on one sample in order to assess the intra-individual coefficient of variation. Erroneous data were further avoided by the checking and confirmation of manual data input by a second individual. Consistency during the analysis procedure was additionally upheld through the usage of constant μCT analysis parameters; only differing in the threshold used for total and cortical bone analysis.

**Table 1:** Bone microarchitecture and density parameters analyzed during total and cortical analysis.

Parameter	Abbreviation (units)	Total analysis	Cortical analysis	Quantitative analysis	Qualitative analysis
Total volume	TV (mm^3^)	Yes	Yes	Yes	–
Bone volume	BV (mm^3^)	Yes	Yes	Yes	Yes
Bone volume Fraction	BV/TV (%)	Yes	Yes	Yes	–
Cortical porosity	Ct.Po (%)	–	Yes	Yes	Yes
Cortical thickness	Ct.Th (mm)	–	Yes	Yes	Yes
Tissue mineral density	TMD (mgHA/cm^3^)	Yes	Yes	Yes	–
Bone surface area	BS (mm^2^)	Yes	Yes	Yes	Yes
Bone surface fraction	BS/BV (mm^−1^)	Yes	Yes	Yes	–

### Statistical Analysis

Statistical analysis was conducted using SPSS v.20 (IBM). A Kruskal–Wallis test was performed on the four groups. To test the effects of spaceflight, spacecraft habitat and recovery, Mann–Whitney *U* tests with Bonferroni’s adjustment were performed between groups and were taken into account when the Kruskal–Wallis test was significant (*p* < 0.05).

## Results

### Body Mass

Launch body mass (**Table 2**) was similar between the groups. A body mass decrease was observed in the Recovery group after landing (−2.62g vs. landing; *p* = 0.043).

**Table 2:** Body mass of the experimental groups.

Parameters	Control Mean (±SE)	Habitat control Mean (±SE)	Flight Mean (±SE)	Recovery Mean (±SE)
Launch body mass (g)	26.05 (±0.41)	27.63 (±0.51)	26.96 (±0.89)	27.68 (±1.09)
Landing body mass (g)	27.71 (±0.56)	27.85 (±0.59)	29.30 (±1.11)	25.06 (±1.05)
Delta body mass at landing (g)	1.66 (±0.26)	0.22 (±0.74)	2.34 (±1.29)	−0.98 (±2.28)
Delta body mass after recovery (g)	_	_	_	− 2.62 (±1.06)

### Reliability of Ankle μCT Parameters

**FIGURE 2:**
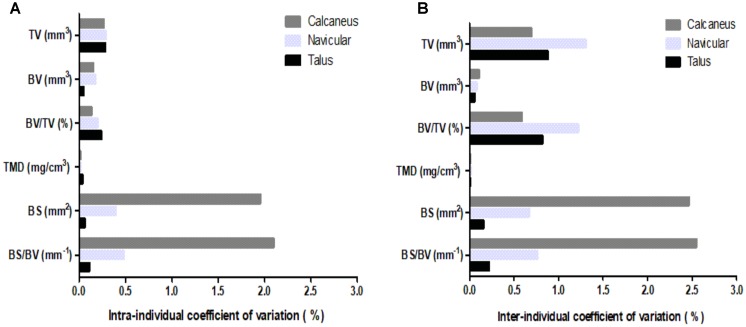
Coefficients of variation for μCT bone parameters (TV, BV, BV/TV, TMD, BS, BS/BV) of the three analyzed ankle bones (calcaneus, navicular, and talus): **(A)** intra-individual CV; **(B)** inter-individual CV.

Coefficients of variation (CV) for intra- and inter-individual analyses (**Figure 2**) were calculated for the six μCT bone parameters, in total bone analyses of all three analyzed ankle bones. The talus displayed the lowest intra-individual variability (**Figure 2A**), 0.1–0.3%, across parameters. The lowest intra-individual CV existed for TMD of all bones; whereas, BS and BS/BV presented with the highest intra-individual variability for the navicular (CV = 0.5%) and particularly the calcaneus (CV = 2%). Nevertheless, intra-individual CVs were low for all parameters.

Inter-individual measurements (**Figure 2B**) presented with greater variability than intra-individual measurements, albeit CVs remained low. The navicular analyses generally showed greatest variability in the region of 1%; although for BS and BS/BV parameters the calcaneus exhibited a greater CV in the region of 2.5%. Similarly, to intra-individual analysis, the talus displayed the lowest CV for four of the six parameters.

### Spacecraft Housing Did Not Alter Bone Ankle Parameters

A comparison between the two control groups (Control and Habitat Control) was performed to estimate the impact of spacecraft housing. No significant difference were found between Control and Habitat Control groups (**Tables 3–5**) indicating that the spacecraft housing did not significantly alter bone ankle parameters in all sites (calcaneus, navicular, and talus).

**Table 3:** Effect of spaceflight, spacecraft housing, and recovery on calcaneus microarchitecture parameters.

Parameters	Control Mean (±SE)	Habitat control Mean (±SE)	Flight Mean (±SE)	Recovery Mean (±SE)
**Whole calcaneus**				
TV (mm^3^)	3.20 (±0.11)	3.12 (±0.1)	2.96 (±0.12)	3.14 (±0.1)
BV (mm^3^)	2.42 (±0.10)	2.26 (±0.1)	2.11 (±0.12)	2.11 (±0.07)
BV/TV (%)	75.46 (±1.04) ^∗^^##^	72.29 (±1.9) ^##^	70.92 (±1.23) ^†^^##^	67.1 (±0.48) ^††^^$$∗∗^
TMD (mgHA/cm^3^)	1039 (±6)	1016 (±9)	1026 (±4)	1027 (±8)
BS (mm^2^)	28.94 (±0.70)	28.63 (±0.73)	27.97 (±0.63)	30.31 (±0.76)
BS/BV (mm^−1^)	11.93 (±0.36) ^##^	12.62 (±0.59)	13.24 (±0.49)	14.25 (±0.18) ^††^
**Cortical calcaneus**				
TV (mm^3^)	2.49 (±0.10)	2.40 (±0.06)	2.22 (±0.12)	2.21 (±0.07)
BV (mm^3^)	2.42 (±0.10)	2.3 (±0.1)	2.16 (±0.11)	2.14 (±0.07)
BV/TV (%)	97.48 (±0.27)	97.11 (±0.32)	97.44 (±0.16)	97.22 (±0.12)
Ct.Po (%)	2.52 (±0.27)	2.89 (±0.32)	2.56 (±0.16)	2.78 (±0.12)
Ct.Th (μm)	171 (±4) ^##^	167 (±6)	163 (±5)	151 (±2) ^††^
TMD (mgHA/cm^3^)	1023 (±6)	998 (±10)	1009 (±4)	1009 (±8)
BS (mm^2^)	28.26 (±0.65)	27.78 (±0.69)	26.74 (±0.12)	28.58 (±0.58)
BS/BV (mm^−1^)	11.73 (±0.31) ^##^	12.05 (±0.46)	12.32 (±0.12)	13.21 (±0.18) ^††^

^∗^*p < 0.05,*^∗∗^*p < 0.01 vs. Flight;*^†^*p < 0.05,*^††^*p < 0.01 vs. Control,*^††^*p < 0.01 vs. Habitat control;*^#^*p < 0.05,*^##^*p < 0.01 vs. Recovery.*

**Table 4:** Effect of spaceflight, spacecraft housing, and recovery on navicular microarchitecture parameters.

Parameters	Control Mean (±SE)	Habitat control Mean (±SE)	Flight Mean (±SE)	Recovery Mean (±SE)
**Whole navicular**				
TV (mm^3^)	1.01 (±0.03)	0.97 (±0.2)	0.94 (±0.04)	1.11 (±0.7)
BV (mm^3^)	0.83 (±0.03)	0.77(±0.3)	0.74 (±0.04)	0.83 (±0.05)
BV/TV (%)	82.01 (±1.03) ^##^	79.83 (±1.49)	78.85 (±0.79)	75.32 (±1) ^††^
TMD (mgHA/cm^3^)	1027 (±4)	1012 (±9)	1008 (±4)	1010 (±10)
BS (mm^2^)	10.94 (±0.34) ^##^	10.85 (±0.29) ^##^	10.98 (±0.42) ^#^	13.12 (±0.55) ^††^^∗^
BS/BV (mm^−1^)	13.14 (±0.53)	14 (±0.71)	14.67 (±0.53)	15.25 (±0.51)
**Cortical navicular**				
TV (mm^3^)	0.89 (±0.03)	0.84 (±0.03)	0.79 (±0.04)	0.88 (±0.05)
BV (mm^3^)	0.86 (±0.03)	0.81 (±0.03)	0.77 (±0.04)	0.85 (±0.05)
BV/TV (%)	97.27 (±0.20)	96.71 (±0.26)	96.85 (±0.15)	96.41 (±0.30)
Ct.Po (%)	2.74 (±0.20)	3.29 (±0.26)	3.15 (±0.15)	3.59 (±0.30)
Ct.Th (μm)	152 (±6)	146 (±7)	136 (±4)	126 (±4)
TMD (mgHA/cm^3^)	1009 (±5)	992 (±10)	1990 (±4)	991 (±10)
BS (mm^2^)	11.53 (±0.36) ^##^	11.20 (±0.27) ^##^	1111.42 (±0.36) ^#^	13.50 (±0.59) ^††^^∗^
BS/BV (mm^−1^)	13.42 (±0.61)	13.80 (±0.65)	114.74 (±0.48)	15.81 (±0.54)

^∗^*p < 0.05 vs. Flight;*^††^*p < 0.01 vs. Control;*^$$^*p < 0.01 vs. Habitat control;*^#^*p < 0.05,*^##^*p < 0.01 vs. Recovery.*

**Table 5:** Effect of spaceflight, spacecraft housing, and recovery on talus microarchitecture parameters.

Parameters	Control Mean (±SE)	Flight Mean (±SE)	Recovery Mean (±SE)
**Whole talus**			
TV (mm^3^)	1.45 (±0.06)	1.46 (±0.06)	1.55 (±0.06)
BV (mm^3^)	1.20 (±0.06)	1.16 (±0.06)	1.18 (±0.06)
BV/TV (%)	82.65 (±1.40) ^#^	79.75 (±1.44)	75.32 (±0.82) ^†^
TMD (mgHA/cm^3^)	1075 (±0.57)	1065 (±9)	1045 (±9)
BS (mm^2^)	13.28 (±0.57)	14.08 (±0.48)	15.74 (±0.31)
BS/BV (mm^−1^)	11.07 (±0.62)	12.03 (±0.51)	13.28 (±0.52)
**Cortical talus**			
TV (mm^3^)	1.25 (±0.07)	1.20 (±0.06)	1.21 (±0.06)
BV (mm^3^)	1.22 (±0.07)	1.18 (±0.06)	1.17 (±0.06)
BV/TV (%)	97.85 (±0.18)	97.67 (±0.19)	97.09 (±0.41)
Ct.Po (%)	2.15 (±0.18)	2.33 (±0.19)	2.91 (±0.41)
Ct.Th (μm)	175 (±12)	162 (±12)	143 (±7)
TMD (mgHA/cm^3^)	1057 (±10)	1048 (±9)	1024 (±10)
BS (mm^2^)	14.14 (±0.62) ^#^	14.63 (±0.51)	16.47 (±0.47) ^†^
BS/BV (mm^−1^)	11.70 (±0.91)	12.39 (±0.50)	13.99 (±0.70)

^†^*p < 0.05 vs. Control;*^#^*p < 0.05 vs. Recovery.*

### Impact of Spaceflight and Reambulation on Calcaneus Bone

Calcaneus microarchitecture parameters are displayed in **Table 3**.

Whole calcaneus BV/TV (%) was decreased in the Flight vs. Control groups (70.92, 75.46; *p* < 0.05). It was also decreased in the Recovery group vs. Flight, Habitat Control and Control groups (67.1, 70.92, 72.29, 75.46, respectively; *p* < 0.01) and BS/BV (mm^−1^) was increased in the Recovery vs. Control groups (14.25, 11.93; *p* < 0.01).

Cortical thickness (μm) was decreased in the Recovery vs. Control groups (151, 171, respectively; *p* < 0.01) and cortical BS/BV was increased (13.21, 11.73; *p* < 0.01). Other parameters were not significantly affected.

**FIGURE 3:**
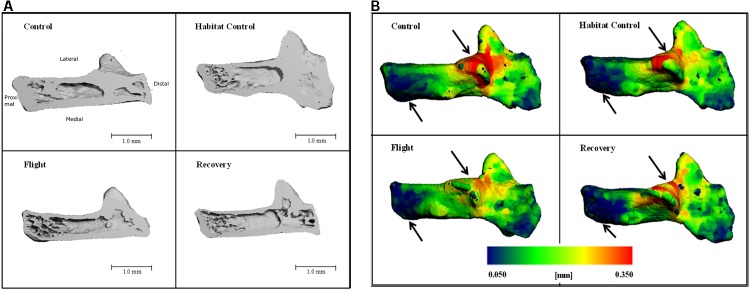
Three-dimensional reconstructions of the calcaneus, illustrating **(A)** cross-section through 3D reconstructions of calcaneus: Control; Habitat control; Flight; Recovery. **(B)** Color maps for Ct.Th of calcaneus; blue = thinnest (0.050 mm) and red = thickest (0.350 mm) for: Control; Habitat control; Flight; Recovery. Black arrows indicate areas of greatest change at articulations.

Cross-sections through the 3D reconstructions of the calcaneus qualitatively indicated a loss of bone volume particularly evident at the proximal end of calcaneus (**Figure 3A**). Further qualitative analysis of the cortical bone was performed utilizing color maps to visualize where Ct.Th changes occurred. **Figure 3B** highlights a decrease in thickness (red areas), predominantly between Control and Flight, and an increase in thinnest blue areas, predominantly between Control and Recovery. These two areas showing greatest cortical thinning correspond with the sub-talar articulation and the point of ground contact.

### Impact of Spaceflight and Reambulation on Navicular Bone

Navicular microarchitecture parameters are displayed in **Table 4**.

Whole navicular parameters tended to be affected across the groups for Flight as compared to Habitat Control and Control groups but differences did not reach statistical significance.

The whole navicular BV/TV (%) was decreased in the Recovery group vs. Control groups (75.32, 82.01; *p* < 0.01) and BS (mm^2^) was significantly increased in the Recovery vs. Flight, Habitat Control, and Control groups (13.12, 10.98, 10.85, 10.94; respectively; *p* < 0.05).

Of note, cortical BS/BV (mm^−1^) were similar between Control (13.42), Habitat Control (13.80), and Flight (14.74), but tended to increase in Recovery (15.84) when compared to all other groups (*p* = 0.08).

**FIGURE 4:**
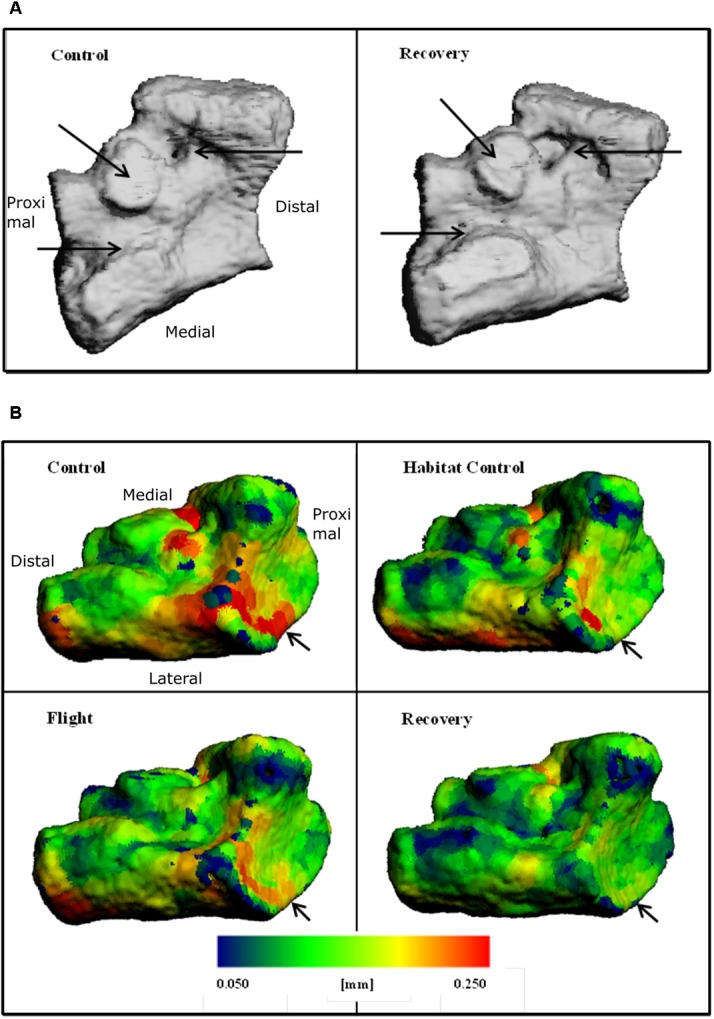
Three-dimensional reconstructions of the navicular, illustrating **(A)** BS and regions of erosion (indicated via black arrows) for Control and Recovery and **(B)** color maps for Ct.Th, blue = thinnest (0.050 mm) and red = thickest (0.250 mm) for: Control; Habitat control; Flight; and Recovery. Black arrows indicate areas of greatest change at articulations.

Increased bone surface area is observable on 3D reconstructions as erosion; black arrows on **Figure 4A** emphasize regions of large erosion between Control and Recovery, in addition to an uneven and irregular surface apparent on Recovery. Color maps of the navicular allow visualization of the decline in Ct.Th across groups (**Figure 4B**). Note the increase in thinnest blue areas (0.050 mm) and the decrease in thickest red areas (0.250 mm), particularly evident between Control and Recovery. Arrows indicate that the site of the talonavicular articulation shows the most dramatic change in Ct.Th.

### Impact of Spaceflight and Reambulation on Talus Bone

**FIGURE 5:**
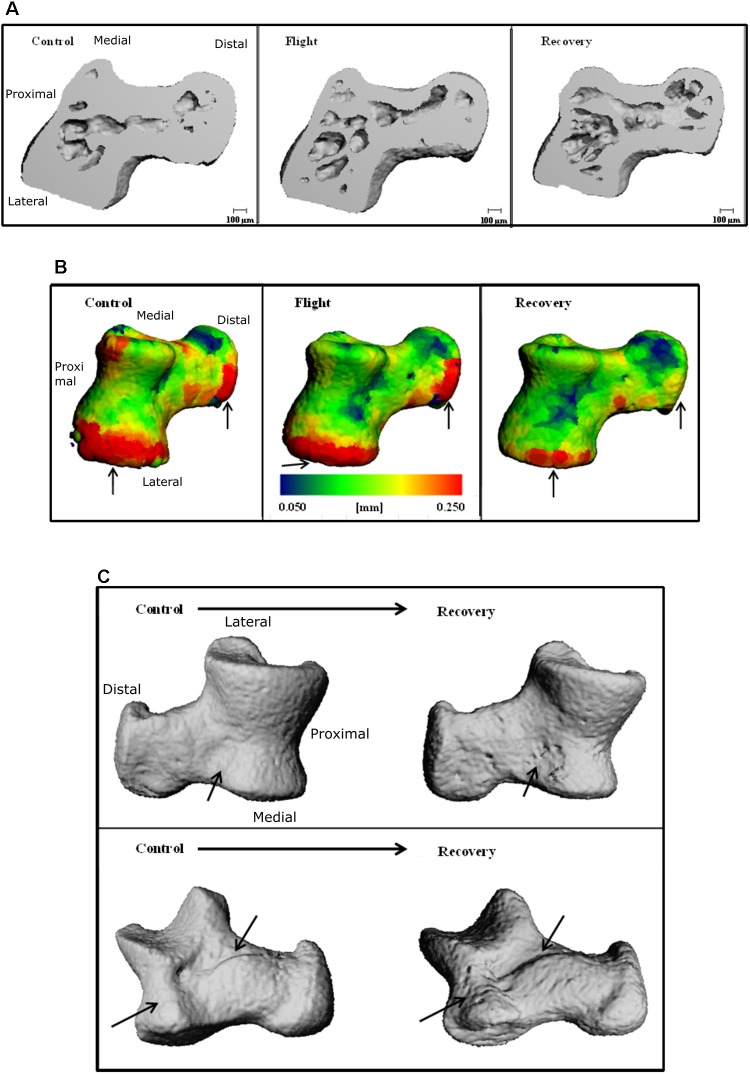
Three-dimensional reconstructions of the talus, illustrating **(A)** cross-section through 3D reconstructions of talus: Control; Flight; Recovery. **(B)** Color maps for Ct.Th of talus blue = thinnest (0.050 mm) and red = thickest (0.250 mm) for: Control; Flight; Recovery. Arrows indicate areas of greatest changes at articulations **(C)** BS regions of erosion (indicated via arrow), for the talus in two positions: (Upper) control and recovery cranial aspect; (Down) control and recovery; recovery underside aspect **(C)**.

Talus microarchitecture parameters are displayed **Table 5**. The whole talus BV/TV (%) was decreased in the Recovery group vs. Control groups (75.3, 82.6; *p* < 0.05) and cortical BS (mm^2^) was significantly increased in the Recovery vs. Control groups (16.47, 14.14; *p* < 0.05). **Figure 4A** illustrated the regions of erosion (indicated via arrows). Further qualitative analysis of Ct.Th utilizing colour maps allowed visualization of local Ct.Th changes. Arrows on **Figure 5B** highlight a decrease in thickest red areas (0.350 mm), predominantly between Control and Flight, and an increase in thinnest blue areas (0.050 mm), predominantly between Control and Recovery. Changes in Ct.Th highlighted by the pointers correspond with the talonavicular and tibiotalar articulations.

## Discussion

Thirty days of microgravity aboard the Bion-M1 mission followed by 8-day recovery induces adaptive site-specific changes in the weight bearing ankle. As these skeletal sites are not usually evaluated, we first assessed the reproducibility of μCT measurements. Results in full bone compartment and in cortical bone compartment were found reproducible (CV included between 0.1 and 2.5%), within coefficients of variation already published by our group in the rat ankle bones (Courbon et al., 2017) and in the standard mouse femur (Kohler et al., 2005). However, bone ankle trabecular compartmental analysis was not robust enough (data not shown) to be considered, due to the poor trabecular network.

Spaceflight induced a mild decrease in bone microarchitecture at all bone sites, the more abundant changes occurring at the calcaneus followed by navicular and talus bones. The whole BV/TV decrease in the three bones (mean −4.59% vs. Control) was much lower than losses we observed in the trabecular compartment of femur or vertebrae of the same mice (−85%, −56% between Flight and Control, respectively) (Gerbaix et al., 2017) and by another group in spinal vertebrae (−20%) (Berg-Johansen et al., 2016). This suggests that more compact ankle bones (total BV/TV from 75 to 83%) were protected from dramatic bone loss, despite their weight bearing function. However, as previously observed in femur and vertebra (Gerbaix et al., 2017), we did not observe, in the ankle bones, any TMD alteration suggesting that the density of the remaining bone was preserved. A μCT analysis of the ischium of 16-week-old female mice exposed to 15-days of microgravity (Blaber et al., 2013) also found a mild, but significant, BV/TV decrease (−6.29%), whilst TMD remained unchanged. However, other studies, using μCT and pQCT, have found significant decreases in TMD alongside BV/TV across multiple bone sites including lumbar spine, femur, and tibia in young male rats after 4-week hind limb unloading or in young female mice after 12-days in space (Jing et al., 2014; Lloyd et al., 2015). These discrepancies of results may be ascribed to disparity in gender, age, and species used across the studies. Both Blaber et al. (2013) and Lloyd et al. (2015) studies utilized female mice, but Lloyd et al. (2015) employed mice that were not skeletally mature.

The μCT quantitative analysis does not indicate spaceflight-induced significant decrease of Ct.Th. However, it is possible that the quantitative analysis alone is misleading as Ct.Th does not present uniformly across the bone. Therefore, the use of qualitative color mapping provided a valuable tool, allowing one to observe Ct.Th changes at local bone sites (Baiker et al., 2012). In doing so, maps suggest that cortical thinning occurs at localized sites, which has not presented as significant across the entire bone due to the averaged nature of the quantitative parameter. Nevertheless, Ct.Th heterogeneity with local erosion transduced in increased specific bone surface area (BS) or bone surface area normalized to bone volume (BS/BV) at the calcaneus and talus. Interestingly, for all bones, the local sites of cortical thinning appeared to correspond with muscle insertions, ground contact, and articulations. The reduced Ct.Th at the muscle insertion could be ascribed to atrophy and or loss of muscular performance occurring in microgravity (Harrison et al., 2003; Shen et al., 2017; Tascher et al., 2017; Radugina et al., 2018). Muscle atrophy has been found to significantly impact the structure of the tendon-bone joint in terms of Ct.Th (Anderson et al., 1993) particularly affecting Ct.Th at the Achilles tendon point of insertion and not at the tibial tuberosity (Johnson et al., 2005). Therefore, it is possible that such an effect is observable at the calcaneus as the anti-gravity soleus muscle is one of the largest atrophying muscles in microgravity (Harrison et al., 2003). Furthermore, it has been found that ankle plantar flexors are the most altered muscle after a 5 weeks bed rest inactivity experience (Berg et al., 2007). This increased proximal-to-distal muscle atrophy would tend to support a similar proximal-to-distal bone loss arguing that distal bone site would be more affected than proximal site as observed in this study for calcaneus bone.

Within the murine model, muscle atrophy leading to cortical thinning appears to occur within short-duration missions of 11-days, inferring that atrophy is rapid in order for the bone geometry changes to occur within the timescale of the current study. The same reason may be attributed to explain the reduction in Ct.Th at the point of ground contact on the calcaneus; but instead with a loss of ground reaction forces rather than muscular load. The explanation for the reduction in Ct.Th at the point of articulation is less clear. Cartilage damage that has been observed in simulated microgravity of the murine model (Niu et al., 2012) results in an impaired ability to prevent articulation friction. Evidence for increased friction can be seen by increased erosion observed at articulation sites (**Figures 4A**, **5C**).

Contrary to our expectation, when reloading occurred on the weight-bearing ankle bones, 8-days of earth reambulation worsened spaceflight-induced bone loss in all bone sites analyzed to a varying extent. After reambulation, bone cortical alterations were more severe in talus compared with calcaneus and navicular bone sites. The recovery group displayed significant decreases in BV/TV and increase in BS in all bone as well as a significant increase in Ct.Th in calcaneus compared to Flight and/or Control group. Similarly in the femur, we did not observe any sign of trabecular rescue whilst cortical bone became thinner (Gerbaix et al., 2017). Several arguments could explain this. Firstly, the decrease of body weight observed between flight and recovery is strongly suggestive of a post-flight stress response, leading to increased cortisol secretion (Ranabir and Reetu, 2011). Hypercortisolism within humans has been associated with increased fracture risk due to reduced bone quality (Chiodini et al., 2009) suggesting that bone microarchitecture is impacted by a stress response of return to Earth. Secondly, previous findings in these Bion-M1 mice showed that spaceflight induced a decrease of the pool of living osteocytes (Gerbaix et al., 2017). Osteocytes orchestrate bone remodeling and their death could lead to bone remodeling imbalance in favor of resorption early post-flight. It was, however, unsurprising that bone quantity, quality, and microarchitecture deterioration worsened during early recovery; since, for the human calcaneus 50% recovery of bone mineral density was mathematically predicted to take up to 9 months (Sibonga et al., 2007) and microarchitecture bone parameters take at least twice the length of spaceflight duration for weight-bearing bone (Vico et al., 2000).

As this is the first study considering microgravity impact on ankle bones of mice, more highly powered studies utilizing μCT or SR-μCT alongside analysis of bone cellular activities would assist in characterizing microarchitectural changes across the foot to identify the region most affected and understand the mechanisms responsible. Studying the muscular load provided by various exercises to the calcaneus may also be imperative for limiting calcaneus bone deterioration.

Overall, spaceflight-induced bone loss is moderated in ankle bones and is both site and compartment specific whilst tissue mineral density of the remaining bone is preserved. Bone erosions and Ct.Th decrease may predominantly appear in articulation, tendon-bone junction, and ground contact sites. Eight days after return from space, bone status worsened as compared to immediate return, which clearly confirmed that bone recovery is a long process and should be taken account considering astronaut support after their return to earth.

## Author Contributions

MG and LV: conceptualization. MG and HW: data curation. MG, HW, and GC: formal analysis. GG-K: funding acquisition. MG, HW, and LV: investigation. GG-K and BS: project administration. LV: supervision.

## Conflict of Interest Statement

The authors declare that the research was conducted in the absence of any commercial or financial relationships that could be construed as a potential conflict of interest.
